# Arginase II Promotes Macrophage Inflammatory Responses Through Mitochondrial Reactive Oxygen Species, Contributing to Insulin Resistance and Atherogenesis

**DOI:** 10.1161/JAHA.112.000992

**Published:** 2012-08-24

**Authors:** Xiu‐Fen Ming, Angana G. Rajapakse, Gautham Yepuri, Yuyan Xiong, João M. Carvas, Jean Ruffieux, Isabelle Scerri, Zongsong Wu, Katja Popp, Jianhui Li, Claudio Sartori, Urs Scherrer, Brenda R. Kwak, Jean‐Pierre Montani, Zhihong Yang

**Affiliations:** 1 From the Laboratory of Vascular Biology, Department of Medicine, Division of Physiology, Faculty of Science, University of Fribourg, Switzerland (X.-F.M., A.G.R., G.Y., Y.X., J.M.C., J.R., I.S., Z.W., K.P., J.-P.M., Z.Y.); 2 Department of Intensive Care Medicine, University Hospital Center and Faculty of Biology and Medicine, Lausanne, Switzerland (J.L.); 3 Department of Internal Medicine, Centre Hospitalier Universitaire Vaudois, Lausanne, Switzerland (C.S.); 4 Department of Cardiology, University Hospital, Bern, Switzerland (U.S.); 5 Facultad de Ciencias, Departamento de Biología, Universidad de Tarapacá, Arica, Chile (U.S.); 6 Department of Pathology and Immunology, Department of Internal Medicine–Cardiology, University of Geneva, Switzerland (B.R.K.); 7 Dr Li is currently affiliated with the Department of Hepatobiliary and Pancreatic Surgery and Centre of Organ Transplantation, The First Affiliated Hospital, Zhejiang University School of Medicine, Hangzhou, China (J.L.)

**Keywords:** atherosclerosis, diabetes mellitus, type 2, inflammation, macrophages

## Abstract

**Background:**

Macrophage‐mediated chronic inflammation is mechanistically linked to insulin resistance and atherosclerosis. Although arginase I is considered antiinflammatory, the role of arginase II (Arg‐II) in macrophage function remains elusive. This study characterizes the role of Arg‐II in macrophage inflammatory responses and its impact on obesity‐linked type II diabetes mellitus and atherosclerosis.

**Methods and Results:**

In human monocytes, silencing Arg‐II decreases the monocytes’ adhesion to endothelial cells and their production of proinflammatory mediators stimulated by oxidized low‐density lipoprotein or lipopolysaccharides, as evaluated by real‐time quantitative reverse transcription‐polymerase chain reaction and enzyme‐linked immunosorbent assay. Macrophages differentiated from bone marrow cells of Arg‐II–deficient (Arg‐II^−/−^) mice express lower levels of lipopolysaccharide‐induced proinflammatory mediators than do macrophages of wild‐type mice. Importantly, reintroducing Arg‐II cDNA into Arg‐II^−/−^ macrophages restores the inflammatory responses, with concomitant enhancement of mitochondrial reactive oxygen species. Scavenging of reactive oxygen species by *N*‐acetylcysteine prevents the Arg‐II–mediated inflammatory responses. Moreover, high‐fat diet**–**induced infiltration of macrophages in various organs and expression of proinflammatory cytokines in adipose tissue are blunted in Arg‐II^−/−^ mice. Accordingly, Arg‐II^−/−^ mice reveal lower fasting blood glucose and improved glucose tolerance and insulin sensitivity. Furthermore, apolipoprotein E (ApoE)–deficient mice with Arg‐II deficiency (ApoE^−/−^Arg‐II^−/−^) display reduced lesion size with characteristics of stable plaques, such as decreased macrophage inflammation and necrotic core. In vivo adoptive transfer experiments reveal that fewer donor ApoE^−/−^Arg‐II^−/−^ than ApoE^−/−^Arg‐II^+/+^ monocytes infiltrate into the plaque of ApoE^−/−^Arg‐II^+/+^ mice. Conversely, recipient ApoE^−/−^Arg‐II^−/−^ mice accumulate fewer donor monocytes than do recipient ApoE^−/−^Arg‐II^+/+^ animals.

**Conclusions:**

Arg‐II promotes macrophage proinflammatory responses through mitochondrial reactive oxygen species, contributing to insulin resistance and atherogenesis. Targeting Arg‐II represents a potential therapeutic strategy in type II diabetes mellitus and atherosclerosis. **(*J Am Heart Assoc*. 2012;1:e000992 doi: 10.1161/JAHA.112.000992.)**

## Introduction

Macrophage‐mediated chronic inflammation is an important mechanism for pathogenesis of obesity‐associated insulin resistance, type II diabetes mellitus, and atherosclerosis.^1–5^ Studies in recent years have provided evidence that macrophages are heterogeneous and undergo heterogeneous phenotypic changes, depending on microenvironmental stimuli.^1,6^ M1 and M2 macrophages are 2 extremes of the cell phenotype spectrum.^6^ M1 macrophages can be induced by microbial agents like lipopolysaccharides (LPS), oxidized low‐density lipoproteins (oxLDL), and T helper cell type 1 (Th1) cytokines (eg, interferon‐γ). The M1 phenotype is associated with increased production of chemoattractants, such as monocyte chemoattractant protein (MCP) 1, and of numerous proinflammatory cytokines, including interleukin (IL) 6 and tumor necrosis factor (TNF) α, and is linked to inflammatory diseases, such as obesity‐associated insulin resistance and atherosclerosis.^6^ In contrast, M2 macrophages are triggered by the Th2 cytokines (eg, IL4 or IL13), are characterized by the production of antiinflammatory cytokines such as IL10, and are considered to participate in resolution of the inflammatory responses.^7,8^

Evidence recently has been presented that reactive oxygen species (ROS) such as superoxide anion (O_2_^•−^) and hydrogen peroxide (H_2_O_2_), especially mitochondrial ROS, are important signals that provoke elevated production of proinflammatory cytokines in monocytes/macrophages.^9–11^. Mitochondrial O_2_^•−^ is converted to H_2_O_2_ by mitochondrial superoxide dismutase‐2, and H_2_O_2_ is further catalyzed to H_2_O and O_2_ by catalase.^12^ ROS are defense mechanisms and play an essential role in immune responses to pathogens, but they also contribute to the pathophysiology of inflammatory diseases, including type II diabetes mellitus and atherosclerosis.^13,14^

Among other genes, arginase has been demonstrated to be involved in macrophage inflammatory responses through regulation of inducible nitric oxide synthase (iNOS).^15^ The underlying mechanism is attributed to depletion of specific intracellular l‐arginine pools for NOS by metabolizing l‐arginine to l‐ornithine and urea.^15^ There are 2 isoenzymes of arginase, arginase I (Arg‐I) and arginase II (Arg‐II), which are encoded by different genes.^16^ Whereas Arg‐I is a cytoplasmic enzyme and primarily functions in the urea cycle involved in ammonia detoxification in the liver, Arg‐II is located in mitochondria and is widely expressed in extrahepatic tissues, particularly the kidney.^16^ Macrophages express both Arg‐I and Arg‐II.^17^ Numerous studies have shown that the enhanced expression of Arg‐I in M2 cells limits the bioavailability of intracellular l‐arginine for excessive nitric oxide production from iNOS and dampens inflammation and tissue damage,^18–20^ contributing to the antiinflammatory functions of M2 cells.^21,22^ Specific targeting of Arg‐I in macrophages has been shown to suppress clearance of intracellular pathogens by decreasing nitric oxide production.^23^ In contrast to Arg‐I, only a few studies have attempted to elucidate the role of Arg‐II in macrophage inflammatory responses. One study proposed that Arg‐II plays a role in antiinflammatory responses on the basis of the association between Arg‐II and liver X receptor in macrophages.^24^ Another study observed that bone marrow–derived macrophages subjected to proinflammatory stimuli express Arg‐II exclusively and that the accumulation of Arg‐II–expressing macrophages is associated with advanced atherosclerotic lesions, which suggests a proinflammatory role of Arg‐II in macrophages.^17^ The results from these studies yielded completely opposite conclusions. Moreover, evidence for a functional role of Arg‐II in macrophages is lacking. The effect of Arg‐II gene disruption on macrophage inflammation in vivo in a chronic inflammatory disease model, such as type II diabetes mellitus and atherogenesis, has not been investigated, although treatment of apolipoprotein E–deficient (ApoE^−/−^) mice with nonspecific arginase inhibitors has been shown to reduce atherosclerosis.^25^

Our study is therefore focused on characterization of the functions of Arg‐II in regulation of macrophage inflammatory responses and of the underlying molecular mechanisms, by means of specific genetic approaches. Moreover, the impact of the genetic targeted disruption of Arg‐II on pathogenesis of chronic inflammatory diseases (ie, obesity‐linked type II diabetes mellitus and atherosclerosis) also is studied.

## Methods

### Materials

Reagents were purchased or obtained from the following sources: Normal chow (NC; energy content: 12% fat, 28% protein, and 60% carbohydrate) was purchased from UAR, Epinay sur Orge, France. A high‐fat diet (HF; energy content: 55% fat, 21% protein, and 24% carbohydrate; Harlan Teklad TD 93075) and a high‐cholesterol diet (HC; energy content: 42.6% fat, 15.5% protein, and 41.9% carbohydrate; 1.3% cholesterol; Harlan Teklad TD.02828) were from Harlan, Horst, the Netherlands. LPS, thioglycollate medium, *N*‐acetylcysteine (NAC), 2′,7′‐dichlorofluorescein (H_2_DCF), propidium iodide solution, and anti‐tubulin (T5168) monoclonal antibody were from Sigma (Buchs, Switzerland). Hoechst 33342 Stain was from ThermoFisher Scientific (Wohlen, Switzerland). Recombinant human TNFα was purchased from Brunschwig (Basel, Switzerland). Human oxLDL was a product of INTRACEL and was purchased from Chemie Brunschwig AG (Basel, Switzerland). Anti–Arg‐I antibody (#610708) was from BD Transduction Laboratories (Allschwil, Switzerland), and anti–Arg‐II antibody (sc‐20151) was from Santa Cruz (Nunningen, Switzerland). Anti‐F4/80 (MCA497G) and anti‐CD11c (MCA1369EL) were from AbD Serotec (Düsseldorf, Germany). Rabbit anti‐MMP14 (ab51074) was from Abcam (Cambridge, UK). Rabbit anti‐iNOS (#06‐573) was from Upstate (Luzern, Switzerland). IRDye 800–conjugated goat anti–rabbit IgG F(c) was purchased from LI‐COR GmbH (Bad Homburg, Germany). CFDA‐SE (carboxyfluorescein diacetate, succinimidyl ester), MitoSox, Alexa Fluor 680–conjugated anti–mouse IgG (H+L), Alexa Fluor 488–conjugated anti‐hamster, and Alexa Fluor 546–conjugated anti‐rat and anti‐rabbit IgG were from Invitrogen (Lubioscience, Luzern, Switzerland). Alexa Fluor 647–conjugated anti‐CD11b antibody was from eBioscience (Vienna, Austria).

### Animals

The Arg‐II–deficient mice (Arg‐II^−/−^) were kindly provided by Dr William O'Brien^26^ and were backcrossed to C57BL/6J more than 8 generations. Genotyping was performed by polymerase chain reaction as previously described.^26^ The wild‐type and Arg‐II^−/−^ offspring from hetero/hetero crossing were interbred to obtain wild‐type and Arg‐II^−/−^ mice, respectively, for experiments. Starting at the age of 7 weeks, the male wild‐type and Arg‐II^−/−^ mice were given free access for 14 weeks to either a normal chow (NC) or a high‐fat (HF) diet. A glucose tolerance test (GTT) and an insulin tolerance test (ITT) were performed after 6 hours of fasting in the twelfth and thirteenth weeks of HF diet, respectively. After 14 weeks of HF, animals were euthanized, followed by collection of blood, peritoneal macrophages, bone marrow, and tissues. The isolated tissues either were subjected to immunofluorescence staining or were snap‐frozen in liquid nitrogen and kept at −80°C until processed. The blood samples were collected after 6‐hour starvation.

The ApoE^−/−^ mice (Jackson Laboratory, Bar Harbor, Maine) and Arg‐II^−/−^ mice, both on a C57BL/6J background, were interbred to obtain ApoE^−/−^Arg‐II^−/−^ mice. To accelerate atherosclerotic lesion formation, 10‐week‐old male ApoE^−/−^Arg‐II^+/+^ and ApoE^−/−^Arg‐II^−/−^ mice were fed either the HF diet or a high‐cholesterol (HC) diet for 10 weeks. At 20 weeks of age, animals were anesthetized with ketamine (100 mg/kg IP) and xylazine (10 mg/kg IP), and the entire aorta from the heart to the iliac bifurcation was removed and dissected free from fat and adhering perivascular tissue. The aortic roots were snap‐frozen in Optimal Cutting Temperature compound. The 7‐μm‐thick cryosections of the aortic roots and the entire thoracic‐abdominal aortas were stained with Oil Red O to assess lesion size, and the size of the necrotic core was evaluated by hematoxylin‐eosine staining of paraffin‐embedded sections of the aortic arches. The sizes of the lesion and the necrotic core were quantified with the ImageJ software (US National Institutes of Health). At least 7 equally spaced aortic root cryosections or 4 equally spaced arch‐paraffin sections per mouse were evaluated. The blood samples were collected after overnight starvation. Housing and animal experimentation were approved by the Swiss Federal Veterinary Office.

### Generation of Recombinant Adenoviruses Expressing Murine Arg‐II and Short Hairpin RNA

Generation of recombinant adenovirus (rAd) expressing murine Arg‐II driven by cytomegalovirus (CMV) promoter (rAd/CMV‐Arg‐II) and short hairpin RNA (shRNA) targeting human Arg‐II (hArg‐II) driven by the U6 promoter (rAd/U6‐hArg‐II^shRNA^) was carried out with the Gateway technology (Invitrogen Life Technologies) according to the manufacturer's instructions. rAd/CMV‐bacterial β‐galactosidase (LacZ) and rAd/U6‐LacZ^shRNA^ were used as controls for rAd/CMV‐Arg‐II and rAd/U6‐hArg‐II^shRNA^, respectively. The pCMV6 construct encoding the murine Arg‐II was obtained from Origene. The human Arg‐II targeting sequence for rAd/U6‐hArg‐II^shRNA^ is indicated in boldface below (only the sense strand is shown):

CACC**GCGAGTGCATTCCATCCTGAA**CGAATTCAGGATGGAATG
CACTCGC

### Cell Culture and Adenoviral Transduction of THP‐1 Cells and Bone Marrow–Derived Macrophages

Human umbilical vein endothelial cells were isolated from umbilical cords and cultivated as described.^27^ The human monocytic cell line THP‐1 (American Type Culture Collection) was maintained in RPMI 1640 medium supplemented with 10% heat‐inactivated fetal bovine serum (HIFBS).

Bone marrow–derived macrophages were prepared from bone marrow–derived cells by incubation with 10% HIFBS‐RPMI, supplemented with 20% of L929‐conditioned medium containing macrophage colony–stimulating factor, for 6 to 7 days to induce differentiation. Treatments were carried out after 6 hours of starvation in 0.2% bovine serum albumin (BSA)–RPMI.

For adenoviral transduction of THP‐1 cells**,** 1×10^6^ THP‐1 cells were harvested in 100 μL RPMI without HIFBS, and 400 μL virus was added. After incubation at 37°C for 2 hours with rotation, 2 mL 10% HIFBS‐RPMI was added. At day 4 after transduction, the transduced cells were serum‐starved in 0.2% BSA‐RPMI for 6 hours before the experiments. For adenoviral transduction of bone marrow–derived macrophages, bone marrow–derived macrophages at day 6 or 7 after differentiation were transduced with rAd at a multiplicity of infection of 100 and were further incubated in 10% HIFBS‐RPMI without L929‐conditioned medium for 2 days. Cells were starved in 0.2% BSA‐RPMI for 6 hours, and then they were either treated with 0.1 μg/mL LPS or left untreated for 22 hours.

### Immunoblotting Analysis

Cell extracts were prepared by lysing cells on ice for 15 minutes by the addition of lysis buffer containing 20 mmol/L Tris.HCl, 138 mmol/L NaCl, 2.7 mmol/L KCl, pH 8.0, supplemented with 5% glycerol, 1 mmol/L MgCl_2_, 1 mmol/L CaCl_2_, 1 mmol/L sodium‐o‐vanadate, 20 μmol/L leupeptin, 18 μmol/L pepstatin, 1% NP‐40, 5 mmol/L EDTA, and 20 mmol/L NaF. Cell debris and nuclei were removed by centrifugation at 10 000×*g* for 10 minutes at 4°C. Protein concentration was determined with the Bio‐Rad DC Protein Assay kit, according to the manufacturer's instruction. Extracts (40 μg) were subjected to SDS‐PAGE and were electrophoretically transferred to an Immobilon‐P membrane (Millipore), and the resultant membrane was incubated overnight with the corresponding first antibody at 4°C, with gentle agitation after blocking with 5% skimmed milk. The protein was then decorated with corresponding anti‐mouse (Alexa Fluor 680–conjugated) or anti‐rabbit (IRDye 800–conjugated) secondary antibodies. Signals were visualized with Odyssey Infrared Imaging System (LI‐COR Biosciences). Quantification of the signals was performed in NIH Image 1.62 software.

### Peritoneal Macrophage Harvest

Peritoneal macrophages were obtained and purified as described previously.^28^ Peritoneal macrophages were harvested with or without thioglycollate elicitation (2 mL of 4% aged thioglycollate medium per mouse, 3 to 4 days) for adhesion assay or immunoblotting analysis, respectively. The macrophages then were purified by magnetic cell sorting with CD11b microbeads and LS columns, according to the manufacturer's instructions (Miltenyi Biotec, Bergisch Gladbach, Germany).

### Monocyte and Macrophage Adhesion Assay

The labeling of monocytes/macrophages and the adhesion assay were performed as described previously.^28^ Briefly, the cultured THP‐1 monocytes or purified peritoneal macrophages were labeled with 5 μmol/L of CFDA‐SE in phosphate‐buffered saline (PBS) at 37°C for 8 minutes. The labeling was stopped with 1 mL heat‐inactivated (56°C×30 minutes) fetal calf serum for 1 minute. The labeled macrophages (2×10^5^ THP‐1 or 1×10^5^ thioglycollate‐elicited murine peritoneal macrophages) were then added to the activated human umbilical vein endothelial cells that had been treated with 10 ng/mL TNFα overnight in 12‐well plates. After incubation for 15 minutes at 37°C, the cells were washed twice with PBS, and images of adherent monocytes or macrophages were captured under the fluorescent microscope (5 different fields per sample). The number of adherent monocytes or macrophages was counted with the ImageJ software (US National Institutes of Health) by a researcher who was blinded to the experimental protocols.

### Adoptive Transfer

Twenty‐week‐old ApoE^−/−^Arg‐II^+/+^ and ApoE^−/−^Arg‐II^−/−^ mice that had received HC diet during the previous 10 weeks were used for adoptive transfer experiments. Monocytes were purified from the splenocytes of the donor mice by magnetic cell sorting with CD11b microbeads and LS columns, according to the manufacturer's instructions (Miltenyi Biotec, Bergisch Gladbach, Germany). Preparation of splenocytes was performed as described previously.^29^ The purity of monocytes was verified by flow cytometry analysis after 1×10^6^ cells were incubated with Alexa Fluor 647–conjugated anti‐CD11b antibody. The purified monocytes were labeled with cell‐permeable Hoechst 33342 Stain (4 μg/mL, 30 minutes) in 10% HIFBS‐RPMI (1×10^6^ cells/mL). After being washed 3 times, the labeled monocytes were resuspended at 2×10^6^/mL in 2% HIFBS‐PBS. Labeled monocytes (1×10^6^) in 500 μL of 2% HIFBS‐PBS were adoptively transferred by intravenous injection into the tail veins of the recipient mice. Sixty hours after transfer, animals were anesthetized with ketamine (100 mg/kg IP) and xylazine (10 mg/kg IP) and euthanized. The aortic roots were prepared and snap‐frozen in Optimal Cutting Temperature compound. The 7‐μm‐thick cryosections of the aortic roots were fixed with ice‐cold methanol for 20 minutes, followed by counterstaining with propidium iodide solution (5 μg/mL, 5 minutes). The number of adoptively transferred labeled monocytes in atherosclerotic plaques was visualized and counted under LEICA's DMI6000 Confocal microscope. Ten equally spaced aortic root cryosections per mouse were evaluated.

### Tissue Immunofluorescence Staining and Confocal Microscopy

After deparaffinization in xylene, hydration in ethanol, and antigen retrieval in citrate buffer (10 mmol/L, pH 6.5) in a microwave oven, paraffin‐embedded sections (7 μm) were blocked with 2% BSA in PBS for 30 minutes and then were incubated with the first antibodies and subsequently with fluorescence‐labeled secondary antibodies at room temperature, for 2 hours and 1 hour, respectively, followed by counterstaining with 300 nmol/L DAPI (4'6‐diamidino‐2‐phenyl‐indole, dihydrochloride, Invitrogen) for 2 minutes. The immunofluorescence signals were visualized under LEICA's DIM6000 Confocal microscope. The same procedure was applied for immunofluorescence staining of the 7‐μm cryosections of aortic roots, except that the sections were fixed with either 0.4% paraformaldehyde (for anti‐F4/80) or methanol (for anti‐CD11c and anti‐MMP14).

### Real‐Time Quantitative Reverse Transcription Polymerase Chain Reaction Analysis

Total RNA extraction and mRNA expression analysis by 2‐step real‐time quantitative reverse‐transcription polymerase chain reaction (qRT‐PCR) were performed as described previously.^30^ The mRNA expression levels of all genes were normalized to the reference gene glyceraldehyde 3‐phosphate dehydrogenase (GAPDH). The primer sequences are summarized in Table 1.

**Table 1.** Primer Sequences for qRT‐PCR

mF4/80F	TGGCTGCCTCCCTGACTTTC
mF4/80R	CAAGATCCCTGCCCTGCACT
mCD11cF	GGAGGAGAACAGAGGTGCTG
mCD11cR	CACCTGCTCCTGACACTCAA
mMCP1F	AGCACCAGCCAACTCTCAC
mMCP1R	TCTGGACCCATTCCTTCTTG
mTNFαF	GGCAGGTCTACTTTGGAGTCATTGC
mTNFαR	ACATTCGAGGCTCCAGTGAATTCGG
mIL6F	GACAACCACGGCCTTCCCTA
mIL6R	GCCTCCGACTTGTGAAGTGGT
miNOSF	GGCAAACCCAAGGTCTACGTT
miNOSR	TCGCTCAAGTTCAGCTTGGT
mArg‐IIF	CCCCTTTCTCTCGGGGACAGAA
mArg‐IIR	GAAAGGAAAGTGGCTGTCCA
mGAPDHF	ACCCAGAAGACTGTGGATGG
mGAPDHR	ACACATTGGGGGTAGGAACA
mMMP14	from Qiagen #QT01064308
hMCP1F	GATCTCAGTGCAGAGGCTCG
hMCP1R	TGCTTGTCCAGGTGGTCCAT
hTNFαF	CCCAGGGACCTCTCTCTAATCA
hTNFαR	GCTACAGGCTTGTCACTCGG
hIL6F	GGCACTGGCAGAAAACAACC
hIL6R	GCAAGTCTCCTCATTGAATCC
hArgIIF	GGCTGAGGTGGTTAGCAGAG
hArgIIR	CTGGCTGTCCATGGAGATTT

Primer sequences are of mouse (m) or human (h) origin.

### Enzyme‐Linked Immunosorbent Assay

The protein level of proinflammatory mediators in the conditioned medium of RPMI containing 0.2% BSA was evaluated by enzyme‐linked immunosorbent assay with the use of the ELISA MAX Deluxe from BioLegend, according to the manufacturer's instructions (Lucerna Chem AG, Luzern, Switzerland).

### Detection of Mitochondrial O_2_^•−^ and H_2_O_2_ in Bone Marrow–Derived Macrophages

Mitochondrial O_2_^•−^ and H_2_O_2_ levels were detected with fluorescent dyes. For detection of mitochondrial O_2_^•−^, cells were incubated with 5 μmol/L MitoSox in culture medium for 10 minutes. For detection of H_2_O_2_, cells were washed with PBS and incubated with 10 μmol/L 2′,7′‐dichlorofluorescein (H_2_DCF) in PBS for 25 minutes. After washing, images were obtained with a Zeiss fluorescence microscope. The intensity of the fluorescence was quantified by ImageJ software (US National Institutes of Health) and normalized by cell number.

### GTT and ITT

GTT and ITT were performed on mice that had been fasted for 6 hours. For GTT, animals were injected intraperitoneally with glucose (1 g/kg), whereas for ITT, 0.5 U/kg human insulin (Actrapid HM, Novo‐Nordisk) was injected intraperitoneally. Blood glucose was measured at 0, 15, 30, 45, 60, 90, and 120 minutes after intraperitoneal injection by a One‐Touch Glucose Monitoring System Ascensia Contour (Bayer).

### Measurement of Blood Parameters

Blood samples were obtained by cardiac puncture after 6‐hour or overnight starvation for wild‐type and Arg‐II^−/−^ mice or for ApoE^−/−^Arg‐II^+/+^ and ApoE^−/−^Arg‐II^−/−^ mice, respectively. The measurement of the total blood cholesterol, triglycerides, and insulin were performed by Services of the Metabolic Platform at the Metabolic Evaluation Facility (MEF), Faculty of Biology and Medicine, University of Lausanne, Switzerland. Blood glucose was measured by a One‐Touch Glucose Monitoring System Ascensia Contour (Bayer).

### Statistical Analysis

The Kolmogorov‐Smirnov test was used to first determine whether the data deviated from Gaussian distributions. For normally distributed values, statistical analysis was performed with the Student *t* test for unpaired observations or analysis of variance with Bonferroni post‐test, and data are given as mean ± standard error of the mean (SEM). For non–normally distributed values, nonparametric statistical analysis was performed with the Mann‐Whitney test or the Kruskal‐Wallis test with Dunn's multiple‐comparison post‐test, and data are expressed as medians with 25th and 75th percentiles. *P*≤0.05 is considered to indicate statistical difference. The “n” indicates the number of individual animals used or the number of individual experiments when conducted with cell lines.

## Results

### Arg‐II Plays a Proinflammatory Role in Macrophages

**Figure 1.**
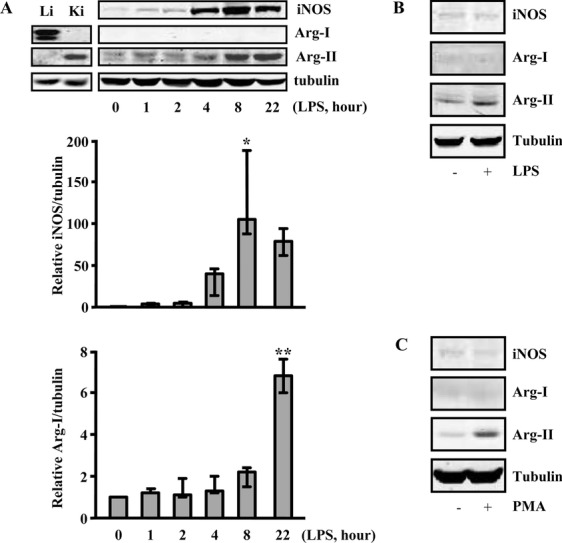
Upregulation of Arg‐II but not of Arg‐I in response to inflammatory activation and differentiation of monocytes/macrophages. A, The murine macrophage cell line RAW264.7 was stimulated with LPS (0.1 μg/mL) for the indicated time points (hours). The expression of iNOS, Arg‐I, and Arg‐II was detected by Western blot. Protein lysates of murine liver (Li) and kidney (Ki) were used as positive controls for Arg‐I and Arg‐II, respectively. Representative blots from 3 independent experiments are shown. The graphs below the blots present the quantification of the signals. Values are medians, and error bars represent 25th and 75th percentiles. The Kruskal‐Wallis test with Dunn's multiple‐comparison post‐test was performed. *P<0.05, **P<0.01 vs control. B and C, The human monocyte cell line THP‐1 was either (B) activated with LPS (0.1 mg/mL, 22 h) or (C) differentiated into macrophages with PMA (phorbol 12‐myristate 13‐acetate; 0.2 μmol/mL, 3 days).

In accordance with previous reports,^31^ we show here that the murine macrophage cell line RAW264.7, when stimulated with LPS (0.1 μg/mL) to induce M1 phenotype activation, revealed an enhanced expression of iNOS and Arg‐II but not Arg‐I (Figure 1A). Similarly, stimulation of the human monocyte cell line THP‐1 with LPS (0.1 μg/mL, 22 hours) upregulated Arg‐II but not Arg‐I (Figure 1B). In contrast to the RAW264.7 cells, no increase in iNOS level was observed in THP‐1 cells (Figure 1B). Moreover, differentiation of THP‐1 monocytes into macrophages by the phorbol ester PMA (phorbol 12‐myristate 13‐acetate; 0.2 μmol/L for 3 days) also was accompanied by increased Arg‐II expression without detectable upregulation of iNOS or Arg‐I (Figure 1C). These results demonstrate that M1 activation and monocyte differentiation to macrophages are accompanied by Arg‐II but not Arg‐I gene upregulation.

**Figure 2.**
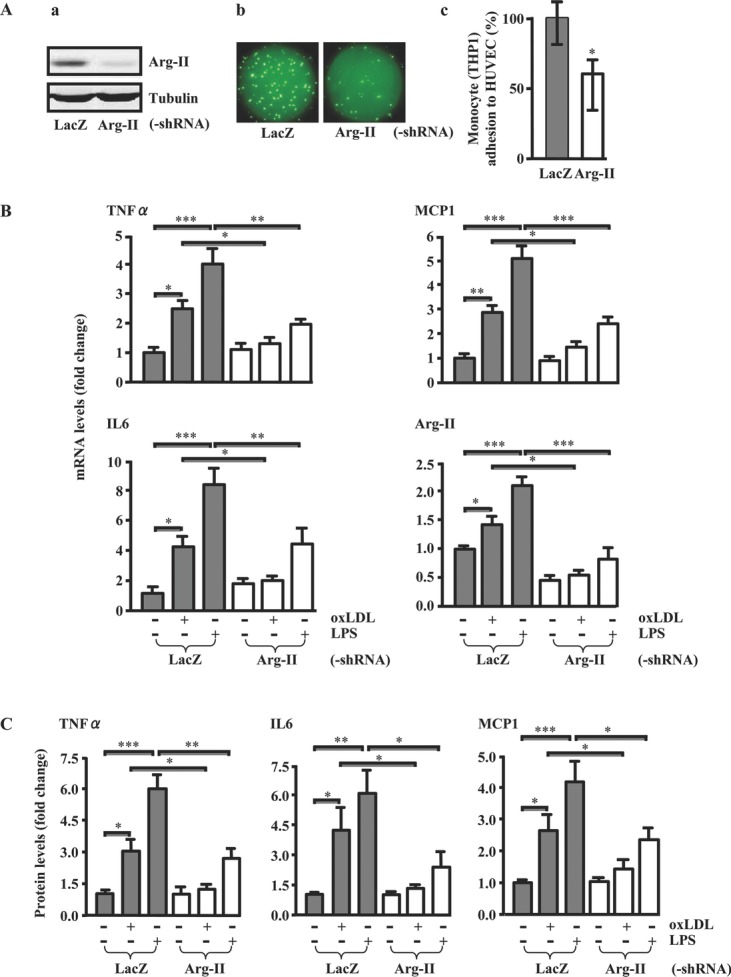
Silencing Arg‐II suppressed proinflammatory functions of human monocytes. A, Silencing Arg‐II in THP‐1 monocytes decreased monocyte adhesion onto activated endothelial cells. THP‐1 cells were transduced with rAd/U6‐LacZ^shRNA^ as control or with rAd/U6‐hArg‐II^shRNA^. Experiments were performed at day 4 after transduction. A‐a, Immunoblotting revealed the efficient knockdown of Arg‐II expression. A‐b, Representative images of THP‐1 adhesion onto TNFα‐activated endothelial cells. A‐c, Quantification of the signals in A‐b from 5 independent experiments. Values are medians, and error bars represent 25th and 75th percentiles. HUVEC indicates human umbilical vein endothelial cells. The Mann‐Whitney test was used. B and C, Silencing Arg‐II in human monocytes decreased the expression of proinflammatory genes. THP‐1 cells were transduced with rAd/U6‐LacZ^shRNA^ as control or with rAd/U6‐hArg‐II^shRNA^. At day 4 after transduction, cells were serum‐starved in 0.2% BSA‐RPMI for 6 or 22 h, followed by stimulation with oxLDL (50 μg/mL) or LPS (0.1 μg/mL) for 24 or 8 h, respectively. Extracted RNA and conditioned medium were then subjected to qRT‐PCR (B) and enzyme‐linked immunosorbent assay (C) analysis, respectively. Data shown are mean±SEM from 6 independent experiments. *P<0.05, **P<0.01, and ***P<0.001 between the indicated groups.

To determine whether Arg‐II is involved in proinflammatory functions of macrophages, THP‐1 cells were transduced with rAd carrying shRNA against human Arg‐II (rAd/U6‐Arg‐II^shRNA^), and cell adhesion activity was assayed. Silencing of Arg‐II, confirmed by immunoblotting (Figure 2A‐a), decreased adhesion activity of THP‐1 cells onto human umbilical vein endothelial cells (prestimulated with 10 ng/mL TNFα overnight to enhance endothelial adhesion molecule expression) when compared to control cells transduced with rAd/U6‐LacZ^shRNA^ (Figure 2A‐b and 2A‐c). The results demonstrate that inhibition of Arg‐II decreases monocyte adhesion activity to endothelial cells.

To further investigate the role of Arg‐II in proinflammatory responses of macrophages, we examined the effect of silencing Arg‐II on the production of proinflammatory mediators in response to proinflammatory stimuli. In THP‐1 cells, both oxLDL (50 μg/mL, 24 hours) and LPS (0.1 μg/mL, 8 hours) upregulated mRNA expression of TNFα, MCP1, and IL6 in parallel with upregulation of Arg‐II (Figure 2B). Silencing Arg‐II significantly blunted the upregulation by oxLDL or LPS at both the mRNA (Figure 2B) and protein (Figure 2C) levels, demonstrating an important role of Arg‐II in proinflammatory responses in monocytes/macrophages. Because the mRNA level reflects the protein level of the inflammatory mediators, the proinflammatory responses therefore were assessed by monitoring mRNA levels for further studies.

**Figure 3.**
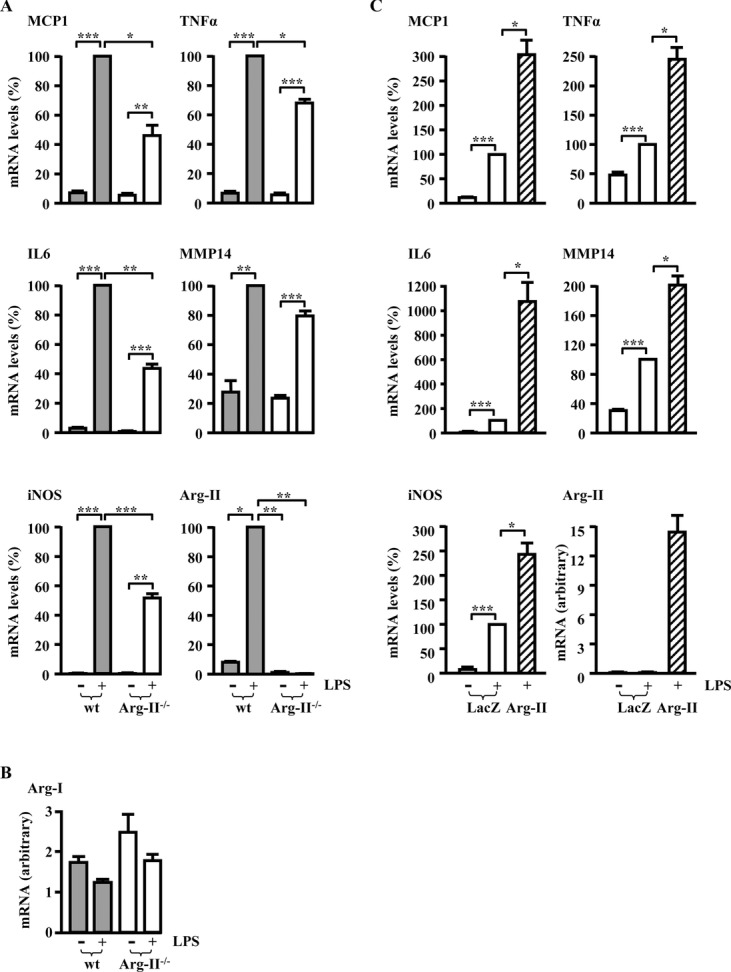
Targeted disruption of Arg‐II suppressed proinflammatory functions of macrophages, and reintroduction of Arg‐II into macrophages from Arg‐II^−/−^ mice restored proinflammatory responses ex vivo. A and B, qRT‐PCR analysis of LPS‐induced (0.1 μg/mL, 22 h) mRNA expression of proinflammatory mediators and Arg‐II (A) as well as Arg‐I (B) in bone marrow–derived macrophages from wild‐type (wt) and Arg‐II^−/−^ mice. Experiments were performed on day 7 after differentiation ex vivo. Cells were serum‐starved for 6 h before addition of LPS. Data are mean±SEM from 12 (untreated groups) or 6 (LPS‐treated groups) individual animals. C, Macrophages differentiated from the bone marrow cells of Arg‐II^−/−^ mice were transduced with rAd/CMV‐LacZ as control or with rAd/CMV‐Arg‐II at day 6 after differentiation ex vivo. Two days after transduction, cells were serum‐starved for 6 h, stimulated with LPS (0.1 μg/mL, 22 h), and subjected to qRT‐PCR analysis. Data shown are mean±SEM from 5 individual animals. *P<0.05, **P<0.01, and ***P<0.001 between the indicated groups.

To reinforce the aforementioned findings, bone marrow cells were isolated from both wild‐type and Arg‐II^−/−^ mice^26^ and were differentiated ex vivo into macrophages. Gene expression of numerous proinflammatory mediators, including MCP1, TNFα, IL6, matrix metalloproteinase (MMP) 14, and iNOS, in response to LPS was reduced significantly in macrophages from Arg‐II^−/−^ mice compared to the cells from wild‐type mice, as assessed by real‐time qRT‐PCR (Figure 3A). The presence and absence of Arg‐II gene expression validate the genotype of the mice (Figure 3A, last graph). Of note, LPS‐induced MMP14 expression was lower in Arg‐II^−/−^ macrophages than in wild‐type macrophages, although the difference was not statistically significant. In agreement with the results from the cell lines shown in Figure 1A through 1C, no upregulation of Arg‐I expression was detected by qRT‐PCR in the macrophages upon LPS stimulation (Figure 3B). Most importantly, reintroduction of mouse Arg‐II cDNA into the macrophages from Arg‐II^−/−^ mice not only restored but also enhanced the LPS‐induced gene expression of all the previously examined proinflammatory mediators, including MMP14, to a much higher level than in the cells from wild‐type mice (Figure 3C). The ectopic expression of Arg‐II was revealed by its mRNA level in the transduced cells (Figure 3C, last graph). These results provide firm evidence for a proinflammatory role of Arg‐II in macrophages.

**Figure 4.**
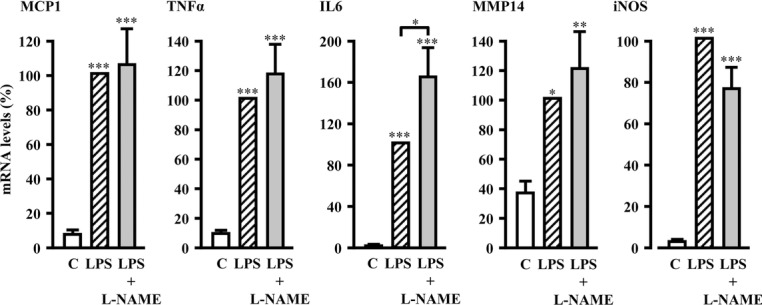
Inhibition of iNOS with L‐NAME affected LPS‐induced production of only IL6 but not of any other proinflammatory mediators in macrophages. qRT‐PCR analysis of LPS‐induced (0.1 μg/mL, 22 h) mRNA expression of proinflammatory mediators in the absence or presence of the NOS inhibitor L‐NAME (5 mmol/L) in bone marrow–derived macrophages from wild‐type mice. Nontreated cells were used as control (C). Experiments were performed on day 7 after differentiation ex vivo. Cells were serum‐starved for 4 h before addition of L‐NAME (pretreatment for 2 h), followed by addition of LPS. Data shown are mean±SEM from 10 individual animals. *P<0.05, **P<0.01, and ***P<0.001 vs control or between indicated groups.

To evaluate the possible role of iNOS in LPS‐induced proinflammatory responses, macrophages from wild‐type mice were treated with L‐N^G^‐nitroarginine methyl ester (L‐NAME;

5 mmol/L), which inhibits all the isoforms of NOS, including iNOS, in macrophages. As shown in Figure 4, inhibition of iNOS did not significantly affect LPS‐induced upregulation of the proinflammatory mediators, including MCP1, TNFα, MMP14, and iNOS itself, except for IL6, which was, however, significantly enhanced by L‐NAME. The results suggest that iNOS does not play a major role in upregulation of the proinflammatory gene expression in macrophages. Rather, it exerts certain antiinflammatory effects in the cells with regard to IL6 expression.

### Arg‐II Promotes Macrophage Proinflammatory Responses Through Mitochondrial ROS

**Figure 5.**
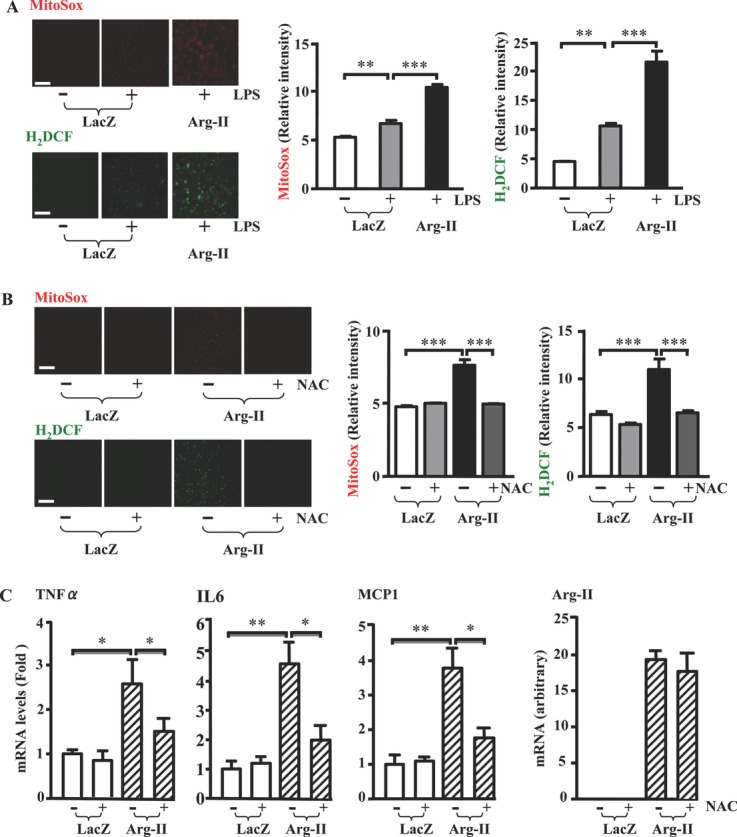
Arg‐II promoted macrophage proinflammatory responses through mitochondrial oxidative stress. A, Bone marrow–derived macrophages were transduced and treated exactly as in Figure 3C and then were subjected to MitoSox or H_2_DCF staining for detection of mitochondrial O_2_^•−^ or H_2_O_2_ levels, respectively. Shown are representative images of experiments performed in macrophages from 5 individual animals. The corresponding bar graphs show the quantification of the relative fluorescence intensity normalized by cell number. Data shown are mean±SEM. B, Bone marrow–derived macrophages from Arg‐II^−/−^ mice were transduced with rAd/CMV‐LacZ as control or with rAd/CMV‐Arg‐II at day 6 after differentiation ex vivo. One day after transduction, cells were treated with NAC (5 mmol/L, 24 h) and then subjected to ROS detection with MitoSox or H_2_DCF staining. Shown are representative images of experiments performed in macrophages from 6 individual animals. The corresponding bar graphs show the quantification of the relative fluorescence intensity normalized by cell number. Data are mean±SEM. C, Bone marrow–derived macrophages from Arg‐II^−/−^ mice were transduced as described in B. NAC (5 mmol/L) was added 12 h after transduction, and cells were incubated for a further 60 h. Cells were serum‐starved in 0.2% BSA‐RPMI during the last 24 h in the absence or presence of NAC as indicated. Total RNA was extracted 72 h after transduction and was subjected to qRT‐PCR analysis. Data shown are mean±SEM from 8 individual animals. Scale bar=50 μm. *P<0.05, **P<0.01, and ***P<0.001 between the indicated groups.

To evaluate the potential mechanism by which Arg‐II promotes macrophage proinflammatory responses, we then examined the effect of Arg‐II on mitochondrial ROS generation. As shown in Figure 5A, the effect of reintroduction of the Arg‐II gene in Arg‐II^−/−^ macrophages on mitochondrial O_2_^•−^ generation and H_2_O_2_ level, as detected by MitoSox and H_2_DCF staining, respectively, coincided with its effect on the proinflammatory responses (Figure 3C). Remarkably, ectopic expression of the Arg‐II gene itself in Arg‐II^−/−^ macrophages, even in the absence of LPS stimulation, enhanced generation of mitochondrial O_2_^•−^ and H_2_O_2_ (Figure 5B), as well as expression of TNFα, IL6, and MCP1 (Figure 5C). Moreover, scavenging both mitochondrial O_2_^•−^ and H_2_O_2_ with *N*‐acetylcysteine (NAC, 5 mmol/L, 60 hours) (Figure 5B) prevented Arg‐II–mediated cytokine production, with no significant effects on the ectopic Arg‐II expression (Figure 5C). These results demonstrate that Arg‐II promotes proinflammatory cytokine production in macrophages through mitochondrial ROS.

### Targeted Disruption of Arg‐II Suppresses Systemic Inflammation in Type II Diabetes Mellitus

**Figure 6.**
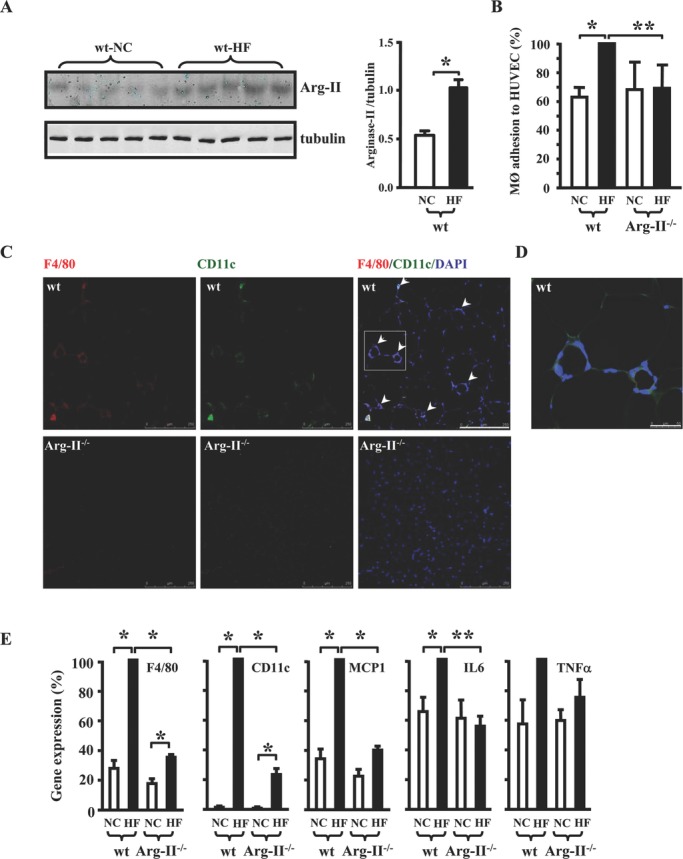
Targeted disruption of Arg‐II suppressed macrophage inflammation in obesity‐linked insulin resistance / type II diabetes mellitus. A, Immunoblotting analysis of Arg‐II expression in peritoneal macrophages of wild‐type (wt) mice fed NC or HF diet. Bar graph shows the quantification of the immunoblots. n=8, *P<0.05 between the 2 groups. B, Quantification of the adhesion assay performed with peritoneal macrophages from 4 different mouse groups as indicated. Data shown are mean±SEM obtained with 5 (NC group) or 6 (HF group) individual mice. HUVEC indicates human umbilical vein endothelial cells. C, Representative confocal images of macrophage infiltration in epididymal fat as assessed by immunofluorescence staining of the paraffin‐embedded sections with antibodies against the pan‐macrophage marker F4/80 (red) and the proinflammatory macrophage marker CD11c (green), followed by nuclei counterstaining with DAPI (blue). Images of F4/80 (left), CD11c (middle), and merged (right) are shown. Scale bar=250 μm. D, Enlargement of the inset in C. Scale bar=50 μm. E, qRT‐PCR analysis of mRNA expression of the inflammatory markers in epididymal fat tissue. Data shown are mean±SEM from 5 (NC group) to 7 (HF group) individual animals. *P<0.05, **P<0.01 between the indicated groups.

To further confirm the role of Arg‐II in macrophage inflammatory responses in vivo, we took advantage of Arg‐II^−/−^ mice. Whole‐body inflammation was induced by HF feeding for 14 weeks, resulting in the low‐grade inflammation model of obesity‐linked insulin resistance.^32^ Immunoblotting analysis showed significantly increased Arg‐II protein levels in peritoneal macrophages of wild‐type mice fed the HF diet as compared to the levels of wild‐type mice fed the NC diet (Figure 6A). Adhesion assays were performed with peritoneal macrophages isolated from wild‐type and Arg‐II^−/−^ mice fed either the NC or the HF diet. As shown in Figure 6B, macrophages from obese wild‐type mice exhibited significantly increased adhesion activity as compared to those from lean animals, whereas the increase in macrophage adhesion activity upon HF was prevented in Arg‐II^−/−^ mice, which confirms the proinflammatory role of Arg‐II observed in cultured human monocytes shown in Figure 2A.

**Figure 7.**
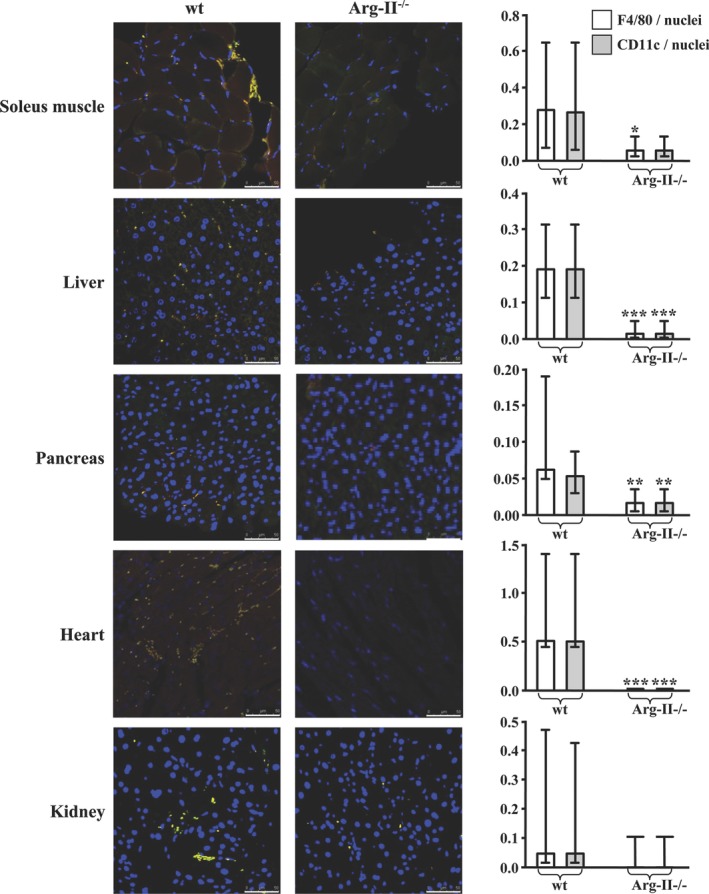
Targeted disruption of Arg‐II suppressed systemic macrophage inflammation in the HF‐induced obesity‐associated insulin resistance mouse model. Representative photos of macrophage infiltration into various tissues, as assessed by confocal immunofluorescence staining of the paraffin‐embedded sections with antibodies against the pan‐macrophage marker F4/80 (red) and the proinflammatory macrophage marker CD11c (green), followed by nuclei counterstaining with DAPI (blue). Shown are the representative merged images from 7 to 11 individual animals of each group. Scale bar=50 μm. Quantification of macrophage infiltration is expressed as a ratio of F4/80‐ or CD11c‐positive cells to tissue nuclei number and is presented in the corresponding graphs on the right. Values are medians, and error bars represent 25th and 75th percentiles. Mann‐Whitney test was used. *P<0.05, **P<0.01, and ***P<0.001 vs wild‐type (wt).

We then investigated whether the HF‐induced chronic inflammation in vivo can be inhibited by Arg‐II gene deficiency. Indeed, the obesity‐associated macrophage infiltration in wild‐type animals on the HF diet, as assessed by confocal microscopic immunofluorescence staining of the pan‐macrophage marker F4/80 and the proinflammatory macrophage marker CD11c, was suppressed in virtually all of the examined organs, including epididymal fat (Figure 6C), soleus muscle, liver, pancreas, heart, and kidney in the Arg‐II^−/−^ mice (Figure 7). The macrophage infiltration in the fat tissue was characterized by typical “crown‐like” structures (Figure 6C and 6D). Moreover, mRNA levels of the macrophage marker F4/80, CD11c, and the inflammatory mediators MCP1 and IL6 in epididymal fat as assessed by qRT‐PCR were increased by HF feeding in wild‐type mice; the increase was prevented or markedly blunted in Arg‐II^−/−^ mice fed the HF diet (Figure 6E). Although TNFα expression tended to be higher in obese wild‐type than in lean wild‐type mice and lower in obese Arg‐II^−/−^ than in obese wild‐type mice, the difference did not reach statistical significance (Figure 6E). These data demonstrate a proinflammatory role of Arg‐II in type II diabetes mellitus in vivo.

### Targeted Disruption of Arg‐II Protects HF‐Induced Insulin Resistance

**Figure 8.**
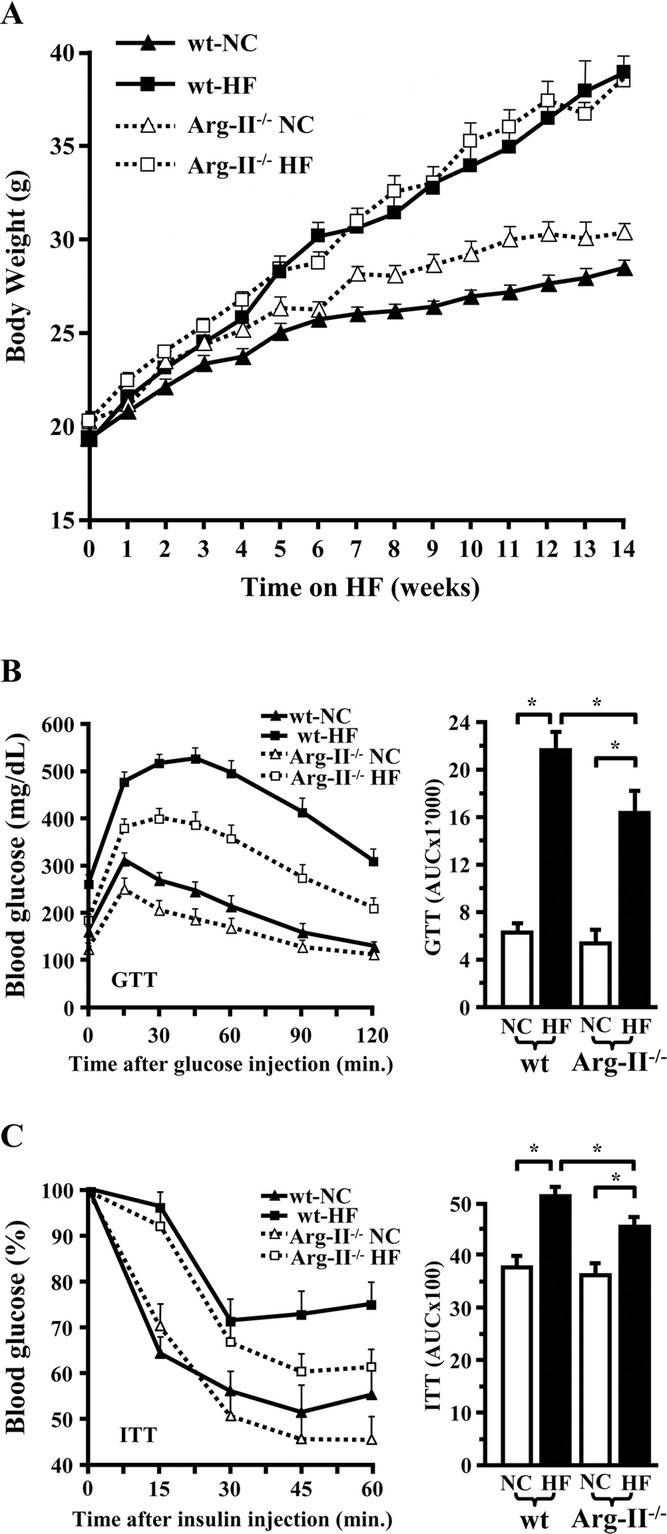
Improved glucose tolerance and insulin sensitivity in Arg‐II^−/−^ mice. A, Growth curve of wild‐type (wt) and Arg‐II^−/−^ mice on NC or HF diets. GTT (B) and ITT (C) in lean and obese wild‐type and Arg‐II^−/−^ mice. Data shown are mean±SEM from 14 to 19 individual animals. Area under the curve (AUC) is presented in the corresponding graphs on the right. *P<0.05 between the indicated groups.

**Figure 9.**
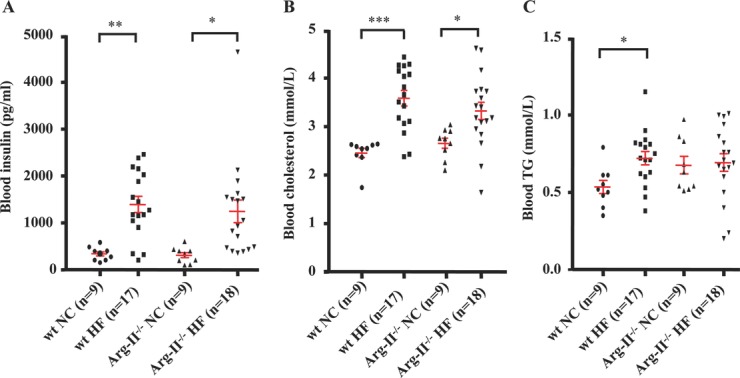
Blood parameters of wild‐type (wt) and Arg‐II^−/−^ mice fed NC (n=9) or HF (n=17 or 18) diets for 14 weeks. Plasma samples were prepared after 14 weeks of HF diet that started at the age of 7 weeks and after 6‐h daytime food withdrawal.*P<0.05, **P<0.01, and ***P<0.001 between indicated groups.

We further investigated whether suppressed chronic inflammation in Arg‐II^−/−^ mice protects against obesity, glucose intolerance, and insulin resistance. There was no significant difference in body weight development between wild‐type and Arg‐II^−/−^ mice fed the HF diet for 14 weeks (Figure 8A). HF feeding increased plasma levels of cholesterol, triglyceride, and insulin to a similar extent in wild‐type and Arg‐II^−/−^ mice (Figure 9). As expected, HF feeding caused hyperglycemia, glucose intolerance, and impaired insulin sensitivity in wild‐type mice, as assessed by GTT and ITT. Glucose tolerance and insulin sensitivity were improved significantly in Arg‐II^−/−^ mice fed the HF diet as compared to wild‐type animals on the HF diet, although they were not completely normalized (Figure 8B and 8C). These results demonstrate that disruption of Arg‐II protects against type II diabetes mellitus independently of obesity.

### Targeted Disruption of Arg‐II Reduces Atherosclerosis

**Figure 10.**
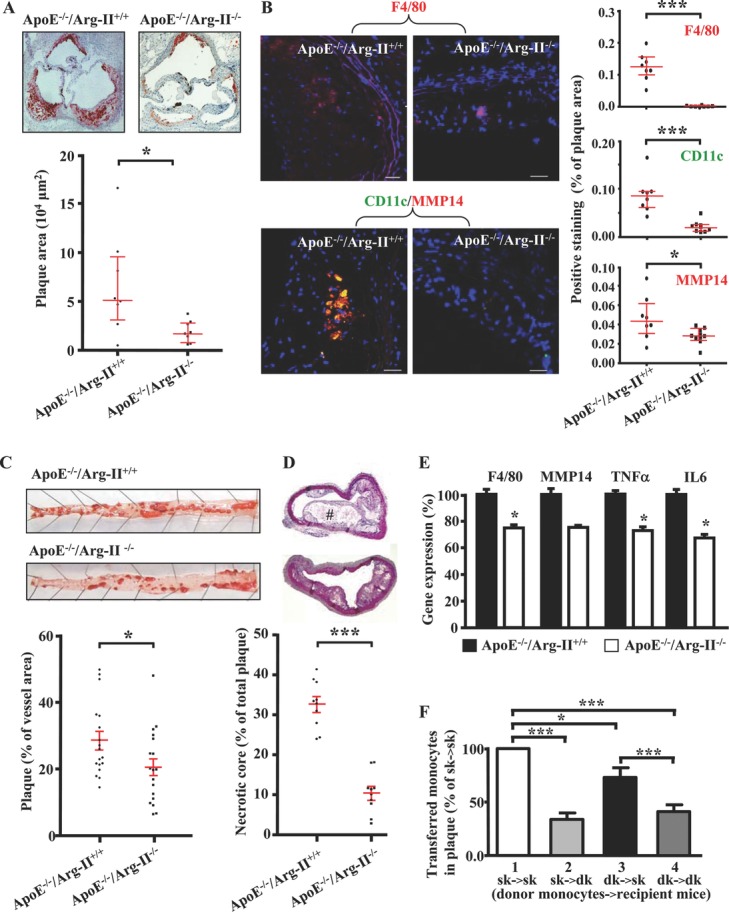
Targeted disruption of Arg‐II reduced atherosclerosis in ApoE^−/−^ mice. Mice were fed either HF (A and B) or HC (C, D, and E) diet for 10 weeks. A, Representative images showing Oil Red O staining of plaques in aortic roots of ApoE^−/−^Arg‐II^+/+^ and ApoE^−/−^Arg‐II^−/−^ mice. Quantifications of the lesions are presented in the graph below the stains. Data shown are medians with 25th and 75th percentiles from 8 animals of each group. At least 7 equally spaced cryosections of aortic roots per mouse were evaluated. B, Representative confocal microscopic images showing macrophage accumulation in the lesions stained with antibodies against F4/80 (red), or CD11c (green) and MMP14 (red). All sections were counterstained with DAPI (blue). The merged images are shown. Scale bars=10 μm. Quantifications of the positive stained cells are presented in the corresponding graphs on the right. Data shown are medians with 25th and 75th percentiles from 8 animals of each group. C, Representative images of Oil Red O staining of lesions in thoracic‐abdominal aortas. Quantifications of the lesions are shown in the graph below the stain (n=18 of each group). D, Hematoxylin‐eosin staining of aortic arches of the 2 mouse groups. Quantifications of necrotic core (indicated by #) are presented as percentage of the cell‐free area to the total lesion area and are shown in the graph below the stain (n=10). At least 4 equally spaced sections per mouse were evaluated. E, qRT‐PCR analysis of F4/80, MMP14, TNFα, and IL6 in the plaques isolated from aortic arches. Data shown are mean±SEM from 10 animals of each group. F, In vivo adoptive transfer. Labeled monocytes from donor ApoE^−/−^Arg‐II^+/+^ (sk: groups 1 and 2) and ApoE^−/−^Arg‐II^−/−^ (dk: groups 3 and 4) mice were injected into recipient ApoE^−/−^Arg‐II^+/+^ (groups 1 and 3: sk→sk and dk→sk, respectively) and ApoE^−/−^Arg‐II^−/−^ (groups 2 and 4: sk→dk and dk→dk, respectively) mice. Data are presented as percentage change to the sk→sk control group. The number of fluorescent macrophages counted in atherosclerotic lesions of the control group (sk→sk) is 61.29±11.46 (n=7 for groups 1 and 3; n=6 for groups 2 and 4). *P<0.05; ***P<0.001 between the indicated groups.

Next, we examined whether the decreased inflammation by Arg‐II gene disruption affects another important chronic inflammatory disease: atherosclerosis.^4,33^ For this purpose, we interbred Arg‐II^−/−^ mice with atherosclerosis‐prone ApoE^−/−^ mice^34^ and obtained ApoE^−/−^Arg‐II^−/−^ mice. To accelerate the atherosclerotic lesion formation, both ApoE^−/−^Arg‐II^+/+^ and ApoE^−/−^Arg‐II^−/−^ mice (at the age of 10 weeks) were fed either the HF diet or an atherogenic HC diet for 10 weeks. The HF diet induced significant atherosclerotic lesions in the aortic roots (Figure 10A) of ApoE^−/−^Arg‐II^+/+^ mice; this was reduced markedly in ApoE^−/−^Arg‐II^−/−^ double‐knockout mice (the lesions in the thoracic and abdominal aortas on HF were too small for quantification, and the data are thus not presented). The proinflammatory macrophage accumulation in the plaque shown by F4/80 staining and CD11c/MMP14 (a vulnerable plaque marker^35^) double‐staining was markedly reduced in ApoE^−/−^Arg‐II^−/−^ mice as compared to ApoE^−/−^Arg‐II^+/+^ controls (Figure 10B). Furthermore, the atherogenic HC diet induced more pronounced lesions through the whole aortas than did the HF diet. The surface lesion size in aortas was significantly smaller in ApoE^−/−^Arg‐II^−/−^ mice than in ApoE^−/−^Arg‐II^+/+^ controls (Figure 10C). In addition, the size of necrotic cores in advanced lesions of the aortic arch was markedly reduced in ApoE^−/−^Arg‐II^−/−^ mice as compared to ApoE^−/−^Arg‐II^+/+^ controls (Figure 10D). qRT‐PCR analysis of plaque samples isolated from aortic arches showed decreased mRNA levels of F4/80, MMP14, TNFα, and IL6 in ApoE^−/−^Arg‐II^−/−^ mice as compared to ApoE^−/−^Arg‐II^+/+^ mice (Figure 10E). No significant difference in plasma cholesterol or triglycerides was observed between the 2 groups (Table 2). These results demonstrate that targeting Arg‐II inhibits atherogenesis and confers features of stable plaques independently of lipid profile.

**Table 2.** Physiological and Plasma Parameters of ApoE^−/−^Arg‐II^+/+^ and ApoE^−/−^Arg‐II^−/−^ Mice Fed HC Diet for 10 Weeks

	ApoE^−/−^Arg‐II^+/+^	ApoE^−/−^Arg‐II^−/−^
Body weight, g		
Begin	24.7±0.5	26.9±0.4*
Final	21.2±0.5	24.3±0.3*
Gain	−3.8±0.8	−2.6±0.3
Total plasma cholesterol, mmol/L	66.9±2.4	65.9±2.5
Plasma triglycerides, mmol/L	0.81±0.04	0.96±0.07

Plasma samples were prepared after 10 weeks of HC diet, starting at the age of 10 weeks and after overnight food withdrawal.

* *P*<0.05 vs ApoE^−/−^Arg‐II^+/+^ (n=18).

Finally, we determined whether circulating monocytes contribute to the reduced macrophage inflammation in the plaque of ApoE^−/−^Arg‐II^−/−^ mice. For this purpose, in vivo adoptive transfer experiments were carried out. In agreement with our in vitro adhesion assay shown in Figure 2A, when ApoE^−/−^Arg‐II^−/−^ double‐knockout (dk) donor monocytes were injected, the number of adoptively transferred monocytes found in the atherosclerotic plaques of ApoE^−/−^Arg‐II^+/+^ single‐knockout (sk) recipient mice was lower, as compared to the control condition in which sk donor monocytes were injected into the same genotypic recipient mice (Figure 10F, dk→sk versus sk→sk). Conversely, recipient dk mice, when compared to recipient sk mice, accumulate fewer donor monocytes of either genotypic mice. (Figure 10F, sk→dk versus sk→sk, and dk→dk versus dk→sk). Of note, no additive reducing effect was observed when both donor monocytes and the recipient mice are deficient in Arg‐II (Figure 10F, dk→dk versus sk→dk). The results demonstrate that both circulating monocytes and the vascular microenvironment in ApoE^−/−^Arg‐II^−/−^ mice contribute to the reduced macrophage inflammation in atherosclerotic lesions in these animals.

## Discussion

In the present study, we provide firm in vitro and in vivo evidence showing that Arg‐II plays a critical role in proinflammatory responses of macrophages. Genetic targeted disruption of Arg‐II in mice suppresses the inflammatory responses, thereby protecting against type II diabetes mellitus, insulin resistance, and atherosclerosis.

The most important novel finding of our present study is the functional characterization of the proinflammatory role of Arg‐II in macrophages. It is widely assumed that Arg‐II shares its function with Arg‐I in macrophages, exerting an antiinflammatory role. However, no experimental evidence has been presented so far, and results obtained from very limited studies on Arg‐II in macrophages are contradictory. A previous study proposed that Arg‐II plays an antiinflammatory role in macrophages,^24^ yet the assumption is based solely on results showing that Arg‐II gene is a direct target of liver X receptor, which has been shown to exert inhibitory effects on expression of inflammatory genes in macrophages. Another recently published study, however, demonstrated that Arg‐II–expressing macrophages correlate with M1 phenotype and are associated with more advanced atherosclerosis.^17^ The results from the later study suggest that Arg‐II could play a proinflammatory role in macrophages. The opposing conclusions of these 2 reports with regard to the role of Arg‐II in macrophage function are based on the correlations. We therefore investigated here the causative effects of Arg‐II on inflammatory responses of macrophages.

We went a step further by using in vitro and in vivo genetic approaches and provided compelling evidence that Arg‐II promotes inflammatory responses in macrophages: (1) M1 activation of macrophages by LPS or differentiation of monocytes into macrophages is accompanied by the upregulation of Arg‐II without having an effect on Arg‐I expression in the cells, which is in agreement with reports from the literature.^31^ Moreover, enhanced Arg‐II expression also is observed in peritoneal macrophages from wild‐type mice fed the HF diet as compared to those of wild‐type mice fed NC, demonstrating a positive association of Arg‐II with proinflammatory responses in macrophages in this chronic inflammation animal model of type II diabetes mellitus. (2) Silencing the Arg‐II gene in human monocyte/macrophage cell lines decreases cell adhesion to endothelial cells and the production of proinflammatory mediators in response to oxLDL or LPS at both the mRNA and protein levels. (3) The adhesion activities of macrophages from HF‐fed Arg‐II^−/−^ mice to endothelial cells also are decreased significantly compared to those from obese wild‐type control mice. It is noticeable that no difference in cell adhesion is observed under NC conditions between wild‐type and Arg‐II^−/−^ mice, which does not seem in line with the observations in the human monocytic cell line (Figure 2A). This might be due to either the species difference^36,37^ or the functional differences between monocytes and macrophages.^38^ Nevertheless, under HF diet conditions, cell adhesion was reduced in Arg‐II^−/−^ mice, which indicates that Arg‐II indeed is involved in monocyte/macrophage adhesion, particularly under pathological conditions. (4) LPS‐induced upregulation of numerous proinflammatory mediators, including MCP1, TNFα, IL6, MMP14, and iNOS, is significantly suppressed in macrophages isolated from Arg‐II^−/−^ mice as compared with those from wild‐type mice. (5) Most importantly, reintroduction of the Arg‐II gene into macrophages from Arg‐II^−/−^ mice restores and even enhances the LPS‐stimulated expression of the proinflammatory genes compared to the levels in cells from wild‐type mice. (6) Ectopic expression of Arg‐II itself in Arg‐II^−/−^ macrophages, in the absence of LPS stimulation, increases expression of the proinflammatory genes. (7) Systemic proinflammatory macrophage infiltration in various organs and expression of proinflammatory mediators in adipose tissues in a chronic inflammation animal model of type II diabetes mellitus are reduced significantly in obese Arg‐II^−/−^ mice. (8) In another chronic inflammation animal model (atherosclerotic ApoE^−/−^ mice), targeted disruption of Arg‐II not only decreases lesion size but also confers characteristics of stable plaque—namely, decreased macrophage accumulation and production of proinflammatory mediators, MMP14, and necrotic cores in the plaque. (9) In vivo adoptive transfer experiments reveal that Arg‐II deficiency in circulating monocytes (ApoE^−/−^Arg‐II^−/−^ donor monocytes) significantly reduces their infiltration into the plaque of recipient ApoE^−/−^Arg‐II^+/+^ mice, reinforcing our observation in the in vitro adhesion assay and indicating that the Arg‐II deficiency in circulating monocytes contributes to the phenotype in ApoE^−/−^Arg‐II^−/−^ mice. The decreased infiltration of donor monocytes from either genotype into the plaque of recipient ApoE^−/−^Arg‐II^−/−^ mice reflects and confirms the fact that there is less inflammation in the vascular microenvironment of the ApoE^−/−^Arg‐II^−/−^ mice than the ApoE^−/−^Arg‐II^+/+^ mice. Collectively, these data provide for the first time in vitro and in vivo novel evidence demonstrating that Arg‐II plays a critical role in proinflammatory responses in monocytes/macrophages.

The function of arginase is considered to be closely related to its involvement in the regulation of nitric oxide production through competition with iNOS for their common substrate l‐arginine, which results in decreased nitric oxide production in macrophages.^39^ iNOS has been considered to play a proinflammatory role in M1 macrophages upon LPS stimulation. Our results, however, do not support a major role of iNOS in mediating proinflammatory responses in macrophages with regard to proinflammatory gene expression. If Arg‐II regulated inflammatory responses through iNOS, inhibition of iNOS with L‐NAME would have a substantial impact on Arg‐II–mediated proinflammatory cytokine gene expression. This is not the case, however. We do not observe significant effects of L‐NAME on the expression of the majority of the proinflammatory genes in macrophages, except IL6, which is significantly enhanced by inhibition of iNOS with L‐NAME. This result is consistent with a previous report^31^ and suggests a negative regulatory role of iNOS on IL6 expression. Moreover, iNOS is not induced by LPS in the human macrophage cell line THP‐1, yet Arg‐II silencing in these cells still inhibits macrophage adhesion activity and expression of the proinflammatory genes. The difference in iNOS expression between human and murine macrophages is in agreement with several other studies.^36,37^ The dissociation of arginase activity from iNOS has been reported by several studies, showing that alteration of arginase activity in macrophages is not necessarily associated with functional changes in iNOS.^40–43^

Evidence has been presented that ROS, especially the mitochondrial ROS, promote production of proinflammatory cytokines.^9–11^ In an attempt to evaluate the molecular mechanisms of Arg‐II–mediated, iNOS‐independent proinflammatory responses in macrophages, we demonstrated that the enhanced mitochondrial ROS are responsible for Arg‐II–mediated proinflammatory responses in macrophages. This conclusion is supported by the following evidence: First, in Arg‐II^−/−^ macrophages, LPS‐induced production of mitochondrial O_2_^•−^ and H_2_O_2_ is markedly enhanced by reintroduction of the Arg‐II gene. In addition, ectopic expression of the Arg‐II gene itself in Arg‐II^−/−^ macrophages, even in the absence of LPS stimulation, also enhances generation of mitochondrial O_2_^•−^ and H_2_O_2._ Second, the production of mitochondrial O_2_^•−^ and H_2_O_2_ parallels the production of proinflammatory cytokines. Third, treatment of bone marrow–derived macrophages with the antioxidant NAC, which abolishes production of mitochondrial O_2_^•−^ and H_2_O_2_, significantly reduces Arg‐II–mediated inflammatory responses. Given that Arg‐II is a mitochondrial enzyme,^44^ a pressing goal for the future will be to investigate how Arg‐II affects mitochondrial function leading to mitochondrial ROS production.

Chronic macrophage inflammation is an important pathogenic mechanism shared by obesity‐linked insulin resistance and atherosclerosis. In support of these notions, we show in the present study that targeted disruption of Arg‐II suppresses macrophage inflammation, contributing to protection against disease development in animal models. In the obesity‐linked insulin resistance model, targeted disruption of Arg‐II in mice improves glucose homeostasis and protects against hyperglycemia in obese mice fed HF diet. It is of note that disruption of Arg‐II does not affect plasma concentration of cholesterol, triglycerides, or insulin, nor does it affect body weight gain, which suggests that Arg‐II ablation protects against type II diabetes mellitus independently of the plasma parameters and obesity, which can be attributed to decreased chronic systemic inflammation in insulin‐responsive tissues. This is in agreement with a previous study reporting that ablation of the proinflammatory macrophages normalizes insulin sensitivity in obese insulin‐resistant animals without affecting body weight gain.^2^

In the atherosclerosis model, genetic ablation of Arg‐II reduces atherosclerotic plaque formation and confers features of stable plaques: reduced lesion size, decreased necrotic core, macrophage accumulation, and proinflammatory mediators in the plaque. The results of genetic ablation of Arg‐II on atherosclerosis development in our present study are in agreement with a recent report by Ryoo et al.^25^ It is important to point out that in their study, a pharmacological approach was taken to assess the effect of an arginase inhibitor on atherosclerosis development in ApoE^−/−^ mice, whereas Arg‐II^−/−^ mice were used to evaluate the role of Arg‐II in atherogenic diet–induced endothelial dysfunction. In our study, we have investigated for the first time the role of Arg‐II in atherosclerosis with a genetic approach, by generating ApoE^−/−^Arg‐II^−/−^ double‐knockout mice. Moreover, we demonstrated that targeting Arg‐II, in addition to preserving endothelial function, also suppresses macrophage inflammation and contributes to reduced atherosclerosis in ApoE^−/−^ mice. Our study thus adds a novel perspective to the linkage between Arg‐II and atherosclerosis.

In summary, our study demonstrates that Arg‐II plays a critical role in macrophage proinflammatory responses through mitochondrial ROS production, contributing to the development of both type II diabetes mellitus and atherosclerosis. Given that general inhibitors of arginase cause deleterious hyperammonemia due to inhibition of hepatic Arg‐I and that no specific inhibitors of extrahepatic Arg‐II are available, our study could open a new therapeutic avenue for developing specific medications targeting Arg‐II for treatment of chronic inflammatory diseases, including type II diabetes mellitus and atherosclerosis.
